# Prognostic significance of EGFR, AREG and EREG amplification and gene expression in muscle invasive bladder cancer

**DOI:** 10.3389/fonc.2024.1370303

**Published:** 2024-05-28

**Authors:** Daniel Uysal, Blerta Thaqi, Alexander Fierek, David Jurgowski, Zoran V. Popovic, Fabian Siegel, Maurice Stephan Michel, Philipp Nuhn, Thomas Stefan Worst, Philipp Erben, Katja Nitschke

**Affiliations:** ^1^ Urologic Research Center, Department of Urology and Urosurgery, Medical Faculty Mannheim, University of Heidelberg, Mannheim, Germany; ^2^ Institute of Pathology, Medical Faculty Mannheim, University of Heidelberg, Mannheim, Germany; ^3^ Department of Biomedical Informatics at the Center for Preventive Medicine and Digital Health, Medical Faculty of Mannheim, University of Heidelberg, Mannheim, Germany; ^4^ Department of Urology, Universitätsklinikum Schleswig-Holstein (UKSH), Kiel, Germany

**Keywords:** EGFR, AREG, EREG, bladder cancer, gene expression, survival

## Abstract

**Introduction:**

Muscle invasive bladder cancer (MIBC) remains a prevalent cancer with limited therapeutic options, obviating the need for innovative therapies. The epidermal growth factor receptor (*EGFR*) is a linchpin in tumor progression and presents a potential therapeutic target in MIBC. Additionally, the *EGFR* ligands *AREG* and *EREG* have shown associations with response to anti-EGFR therapy and improved progression-free survival in colorectal carcinoma.

**Materials and methods:**

We investigated the prognostic significance of *EGFR*, *AREG*, and *EREG* in MIBC. Gene expression and copy number analyses were performed via qRT-PCR on tissue samples from 100 patients with MIBC who underwent radical cystectomy at the University Hospital Mannheim (MA; median age 72, interquartile range [IQR] 64–78 years, 25% female). Results were validated in 361 patients from the 2017 TCGA MIBC cohort (median age 69, IQR 60–77 years, 27% female), in the Chungbuk and MDACC cohort. Gene expressions were correlated with clinicopathologic parameters using the Mann-Whitney test, Kruskal-Wallis- test and Spearman correlation. For overall survival (OS), cancer-specific survival (CSS) and disease-free survival (DFS) gene expression was analyzed with Kaplan-Meier and Cox-proportional hazard models.

**Results:**

Significant gene expression differences in *EGFR*, *AREG*, and *EREG* could be detected in all cohorts. In the TCGA cohort, *EGFR* expression was significantly elevated in patients with EGFR amplification and KRAS wildtype. High *AREG* expression independently predicted longer OS (HR = 0.35, CI 0.19 - 0.63, p = 0.0004) and CSS (HR = 0.42, CI 0.18 – 0.95, p = 0.0378) in the MA cohort. In the TCGA cohort, high *EGFR*, *AREG*, and *EREG* expression correlated with shorter OS (*AREG*: HR = 1.57, CI 1.12 – 2.20, p = 0.0090) and DFS (*EGFR*: HR = 1.91, CI 1.31 – 2.8, p = 0.0008). *EGFR* amplification was also associated with reduced DFS.

**Discussion:**

High *EGFR* and *EREG* indicate worse survival in patients with MIBC. The prognostic role of AREG should further be investigated in large, prospective series. Divergent survival outcomes between the four cohorts should be interpreted cautiously, considering differences in analysis methods and demographics. Further *in vitro* investigations are necessary to elucidate the functional mechanisms underlying the associations observed in this study.

## 1 Introduction

In 2023, bladder cancer (BC) was expected to account for an estimated 82,290 new cases and 16,710 cancer-related deaths in the United States, making it the 4^th^ most common and the 8^th^ most deadly malignancy in men in the US (1). In Europe, BC is the 5^th^ most common cancer with an incidence of more than 200,000 new cases per year (2). While 70% of BC are localized to the mucosa and submucosal tissues and thus classified as non-muscle invasive bladder cancer (NMIBC), 30% invade into the musculature (muscle invasive bladder cancer, MIBC). While the 5-year overall survival (OS) rate for NMIBC is 81%, this drops to 48% when the disease progresses to MIBC (3). Approximately 50% of patients with MIBC develop metastases and have a 5-year survival rate of about 5% (4). Radical cystectomy (RC) with pelvic lymphadenectomy, urinary diversion and in selected cases neoadjuvant chemotherapy, remains the treatment of choice for localized MIBC. In the past decade, treatment in the metastasized setting has evolved from platinum-based chemotherapy regimens to immune checkpoint inhibitors to now include antibody-drug conjugates such as Enfortumab-Vedotin and pan-FGFR inhibitors like Erdafitinib. While these new therapies have the potential to revolutionize the treatment of metastatic MIBC, not all patients will respond and ultimately resistances will arise. Thus, new additional biomarkers and molecular targets in MIBC are still urgently needed. The tyrosine kinase receptor epidermal growth factor receptor (*EGFR*) has been proposed as a potential target in BC, due to its routine therapeutic inhibition in metastatic colorectal carcinoma (mCRC) and non-small cell lung cancer (NSCLC) (5, 6). Immunohistochemistry (IHC) studies in BC showed a comparatively low EGFR expression in normal urothelium and up to 74% protein overexpression in BC, further highlighting the potential role of EGFR as a target in MIBC (7). So far, clinical trials targeting *EGFR* in BC could not convincingly show a benefit for *EGFR*-directed therapy, except for a small group of patients (8, 9). Preclinical studies by Rebouissou et al. support the hypothesis, that a subset of patients with BC will likely respond to *EGFR*-directed therapy (10). Based on these observations, Goodspeed et al. developed a gene signature from *EGFR*-inhibited mCRC data, that could predict the response of BC cell lines to *EGFR* inhibition (11). This signature included, Amphiregulin (*AREG*) and Epiregulin (*EREG)*, two of the seven potential *EGFR* ligands, whose predictive role for *EGFR* inhibition and progression-free survival (PFS) has further been confirmed in mCRC (12–15). Based on these promising preclinical and clinical data, we investigated clinicopathologic and survival associations of *EGFR*, *AREG* and *EREG* gene expression and copy number alterations on OS, disease-free survival (DFS) and cancer-specific survival (CSS) in a cohort of patients with MIBC after RC from Mannheim, and on OS and DFS in a second cohort of patients with MIBC from the The Cancer Genome Atlas (TCGA) project (16). Furthermore, OS and CSS were further analyzed in patients with MIBC from the Chungbuk National University Hospital (17) and the MD Anderson Cancer Center (MDACC) MIBC cohort (18).

## 2 Materials and methods

### 2.1 Patients and methods

**Figure 1:**
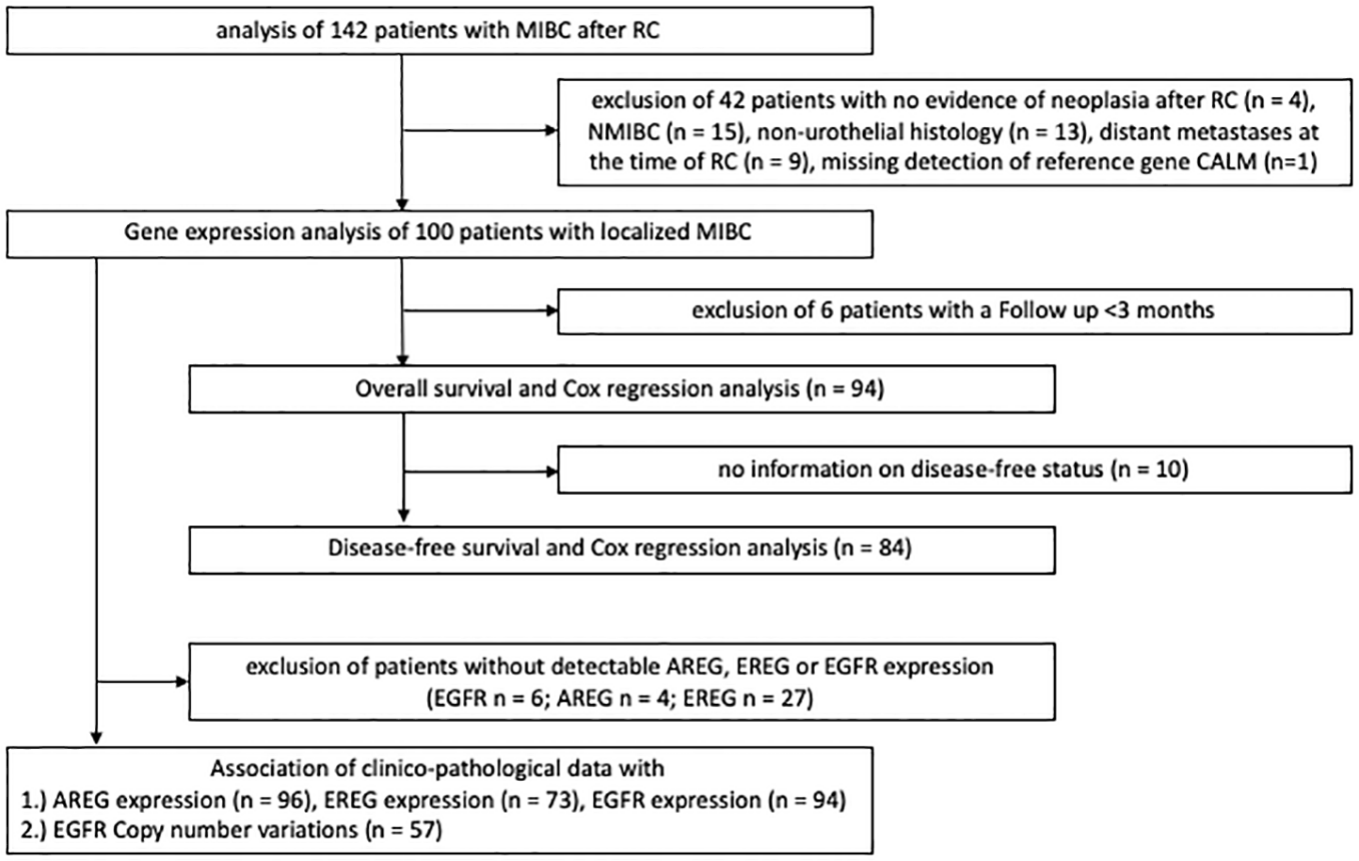
REMARK flow diagram of the exclusion criteria of the Mannheim cohort.

Four independent cohorts were included in this study. The first cohort consisted of patients with MIBC who underwent RC at the Department of Urology and Urosurgery at the University Medical Center Mannheim (MA) between 2008 and 2014. Patients with either no histologic evidence of residual malignancy after RC, NMIBC, non-urothelial histologic subtype, distant metastases at the time of RC or missing detection of the reference gene Calmodulin 2 (*Calm2*) were excluded from further analysis. **Figure 1** shows the different stages of analysis with respective exclusion criteria of the MA cohort. Formalin-fixed paraffin-embedded (FFPE) tissue specimens and clinical data were collected in a retrospective study design which had been approved by the local ethics committee before the study (2015–549N-MA). All participants provided written informed consent. All procedures in this study were carried out in accordance with the 1964 declaration of Helsinki and its later amendments or comparable ethical standards. The other three cohort included patients with MIBC from the TCGA cell 2017 study by Robertson et al. (16), from the Chungbuk National University Hospital study by Kim et al. (17) and from an MDACC cohort by Choi et al. (18).

### 2.2 RNA extraction, cDNA synthesis and quantitative PCR analyses of the gene expressions and copy number alterations in patient samples from the MA cohort

All pathology specimens from the MA cohort were evaluated by the Institute of Pathology of the Medical Faculty Mannheim, University of Heidelberg. Tumor bearing FFPE tissue samples were stained with hematoxylin and eosin, examined by a board-certified uro-pathologist (ZVP) and graded according to the TNM classification (2017) and the WHO 2010/2016 classification of genitourinary tumors. RNA extraction was performed with the magnetic-based XTRAKT FFPE kit (Stratifyer, Cologne, Germany) according to the manufacturer’s instructions. For the quantitative real-time polymerase chain reaction (qRT-PCR), RNA was reversely transcribed into cDNA with Sequence-specific reverse primers (reference gene *Calm2* and target genes *EGFR*, *AREG* and *EREG*), Superscript III reverse transcriptase (Thermo Fisher Scientific, Waltham, MA, USA) and supporting reagents were incubated at 55°C for 120 min with a subsequent enzyme deactivation step at 70°C for 15 min. cDNA was amplified through 40 cycles at 95°C for 3s and 60°C for 30s on a StepOnePlus qRT-PCR cycler (Applied Biosystems, Waltham, MA, USA). *Calm2* was used as a reference gene for normalization and gene expression determined using the 40-(ΔCt)-method (19). **Supplementary Table S1** shows the primers and probes used in this study. Copy number alterations (CNA) in the MA cohort were measured using qPCR with TaqMan Assays according to the manufacturers’ instructions (**Supplementary Table S2**). Predicted CNA values ≥3 were classified as amplification events.

### 2.3 *In silico* validation of findings

**Figure 2:**
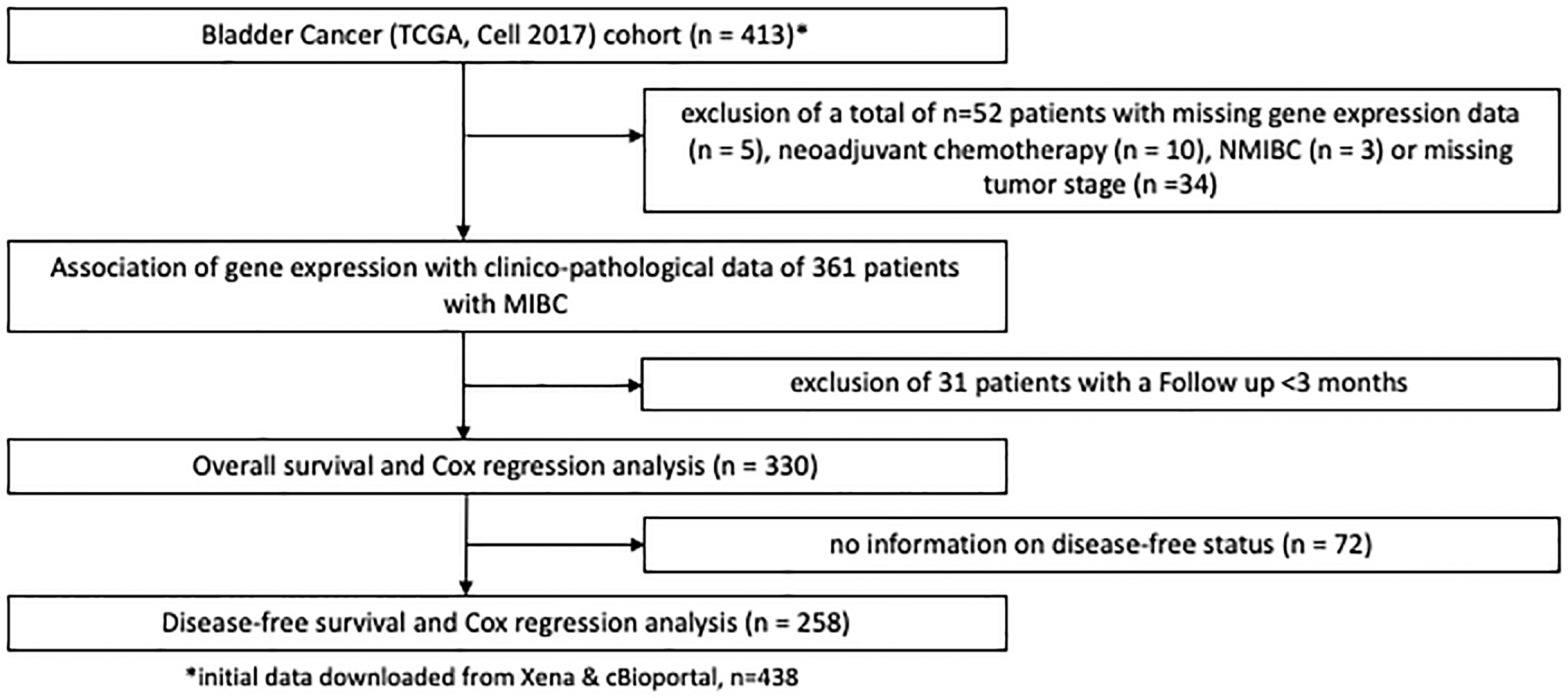
REMARK flow diagram of the exclusion criteria of the second study cohort based on the TCGA cell 2017 MIBC cohort.

Findings were validated in patients with MIBC from the TCGA, the Chungbuk and the MDACC cohort (16–18). **Figure 2** shows patient exclusion criteria at different stages of the analysis of the TCGA cohort. All TCGA data were downloaded from public repositories and have been produced in earlier analyses. CNA and gene expression data were downloaded from the Xenabrowser (https://xenabrowser.net) (20). Clinical data were downloaded from cBioPortal (https://www.cbioportal.org) (21, 22). Briefly, CNA data were generated using Affymetrix SNP6.0 arrays and mRNA gene expression data obtained through RNA Sequencing on an Illumina HiSeq. Data underwent further bioinformatic processing. CNA data were curated with the GISTIC2.0 algorithm and gene expression data quantified and normalized using RNA-Seq by Expectation Maximization (RSEM) and expressed as log_2_. CNA data ranged from -2 to 2, with the following nomenclature applied: -2: 2 copy del; -1: 1 copy del; 0: no change, 1: amplification, 2: high amplification. For the purposes of this study a GISTIC2.0 value of 2, or high amplification defined EGFR amplification. All other gene-level events (-2 to 1) were defined as non-amplification. **Supplementary Figure S1** shows patient exclusion criteria for the Chungbuk and the MDACC cohorts. Similar exclusion criteria were applied as in the MA and TCGA cohort. Clinical and genomic data for the two cohorts were obtained from the Gene Expression Omnibus database (Chungbuk: GSE13507; MDACC: GSE48276) and were included as **Supplementary Material**. Gene expression in the Chungbuk cohort was measured using Illumina Human-6 BeadChip microarrays on RC specimens (17). Gene expression in the MDACC cohort was measured using FFPE TUR-B tissue samples on an Illumina HumanHT-12 WG-DASL V4.0 R2 expression beadchip (18).

### 2.4 Statistical analyses

Statistical analyses were performed using JMP16 (SAS, Cary, North Carolina, USA) and GraphPad Prism 9.0 (GraphPad Software Inc., La Jolla, California, USA). All p values were calculated for two sided tests with p ≤ 0.05 regarded as statistically significant. Statistical analyses of numeric continuous variables following a non-normal distribution were performed with non-parametric tests (Mann-Whitney-Test, Kruskal-Wallis test). Spearman coefficient analysis was performed to assess gene correlations. Survival analyses were performed using the Kaplan-Meier method and differences between subgroups were tested for significance with the log-rank test. Cut-off values for high and low gene expression groups were provided by using the partition test, with each group representing at least 20% of the total cohort. Uni- und multivariable analyses were performed using Cox-proportional hazard regression models, accepting a cut-off value of p < 0.2 to include relevant clinical or pathologic variables into the multivariable analyses, that would have been missed with a more restrictive p value of ≤ 0.05.

## 3 Results

### 3.1 Patient demographics

After exclusion of 42 patients (from initially 142 patients) 100 patients with histologically confirmed urothelial MIBC (median age: 72, range: 64 – 78, 25% female patients, 72% locally advanced carcinomas (T3/4)) remained for the subsequent analyses in the MA cohort. In the TCGA MIBC cohort 361 patients could be evaluated after exclusion criteria were applied (median age: 69, range:60 – 77, 27% female patients, 68% T3/4 tumors). Furthermore, 55 patients with MIBC from the Chungbuk cohort (median age 66, IQR 60–73 years, 20% female) and 38 patients from the MDACC MIBC cohort (median age 68, IQR 60–72 years, 16% female) were analyzed after exclusion criteria (**Supplementary Figure S1**).

Demographic and clinicopathologic data of these cohorts are shown in **Table 1**. Baseline characteristics between the four cohorts were comparable except for a slightly higher percentage of patients with lymph node metastases (N+) in the TCGA cohort. At a median follow-up (f/u) of 39.5 months (range 3 – 180 months, n = 94) 60 patients in the MA cohort died during the f/u period. Of those 60 patients, 31 (52%) died of MIBC. Median f/u among surviving patients was 120 (72.5 – 142) months. In the TCGA cohort median f/u for surviving patients was 24.8 (14.8 – 53.4) months. Median f/u in the original Chungbuk cohort was 37 (1–137) months (17) and in the MDACC cohort median f/u was 45.2 (4 – 180) months.

**Table 1:** Demographics of patients in the Mannheim (MA), the TCGA, the Chungbuk, and the MDACC cohort.

Characteristic	MA cohort (n = 100) n (%)	TCGA cohort (n = 361) n (%)	Chungbuk cohort (n=55) n (%)	MDACC cohort (n=38) n (%)
Age (years)*	72 (64 - 78)	69 (60- 77)	66 (60 – 73)	68 (60 – 72)
Gender				
Male	75 (75%)	264 (73%)	44 (80%)	32 (84%)
Female	25 (25%)	97 (27%)	11 (20%)	6 (16%)
Pathological T-stage				
T2	28 (28%)	117 (32%)	29 (53%)	10 (26%)
T3	55 (55%)	188 (52%)	18 (33%)	21 (55%)
T4	17 (17%)	56 (16%)	8 (15%)	7 (18%)
Lymph node metastases				
negative	73 (76%)	216 (64%)	45 (83%)	17 (45%)
positive	23 (24%)	122 (36%)	9 (17%)	21 (55%)
NA	4	23	1	

* median (range); NA, not available.

### 3.2 Gene expression analysis

**Figure 3:**
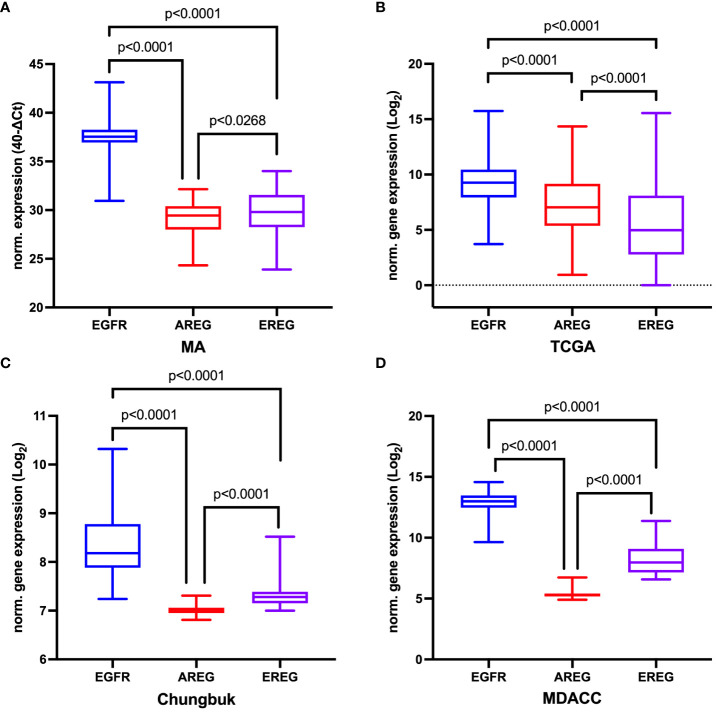
Comparison of normalized gene expression of *EGFR, AREG* and *EREG* **(A)** in the Mannheim cohort, **(B)** in the TCGA cell 2017 MIBC cohort, **(C)** in the Chungbuk cohort, and **(D)** in the MDACC cohort. The p value from the Kruskal-Wallis test between all three genes was p<0.0001. For comparisons between two genes the Mann-Whitney-Test was used.

Overall, *EGFR* showed the highest gene expression across all four cohorts (MA: *EGFR* median CT value 37.54 (range 36.94 - 38.25), median CT value *AREG* 29.45 (range 27.99 – 30.39), median CT value *EREG* 29.80 (range 28.25 – 31.56); TCGA: *EGFR* 9.26 (range 7.94 – 10.45), *AREG* 7.04 (range 5.37 – 9.17), *EREG* 4.98 (range 2.78 – 8.09); Chungbuk: *EGFR* median value 8.18 (range 7.88 – 8.180), *AREG* median 7.01 (range 6.95 – 7.06), *EREG* median value 7.28 (range 7.15 – 7.39); MDACC: *EGFR* median value 12.99 (range 12.47 – 13.47), *AREG* median value 5.32 (range 5.17 – 5.42), *EREG* median value 7.97 (range 7.14 – 9.09), **Figure 3**). Comparing the median gene expression between *EGFR*, *AREG* and *EREG* revealed significant differences between the three genes across all cohorts (p < 0.0001 for all cohorts, **Figure 3**). *AREG* and *EREG* were inversely related between the two cohorts, with a higher *AREG* expression in the TCGA cohort, while AREG showed a lower expression compared to EREG in the other three cohorts. Although high amplification (AMP), compared to low or no amplifications (NOT) of *EGFR* in the TCGA cohort, resulted in a higher gene expression in all three genes, only *EGFR* reached a statistically significant difference (*EGFR*: AMP 13.38 (range 12.08 – 14.66) vs. NOT 9.11 (range 7.89 – 10.21); p < 0.0001, **Supplementary Figure S2A**). Among the 57 patients from the MA cohort with available CNA information on *EGFR* only marginal, non-significant differences in gene expression between the two groups could be observed (AMP vs. Not, **Supplementary Figure S2B**). Assessing the correlation between the three genes revealed a moderately positive correlation between *AREG* and *EREG* in the MA cohort (Spearman ρ = 0.4960, p < 0.0001) and a highly positive correlation in the TCGA cohort (Spearman ρ = 0.7333, p < 0.0001). *EGFR* and both of its ligands were weakly correlated across both cohorts (**Supplementary Figure S3**). In the other cohorts, *AREG* and *EREG* were only weakly correlated (Chungbuk: Spearman ρ = 0.0091, p = 0.9472; MDACC: Spearman ρ = 0.0594, p = 0.7231). In the MDACC cohort, *EGFR* and *EREG* showed a weak positive correlation (Spearman ρ = 0.3457, p = 0.0335) (**Supplementary Table S3**).

### 3.3 Association with clinicopathologic data

**Figure 4:**
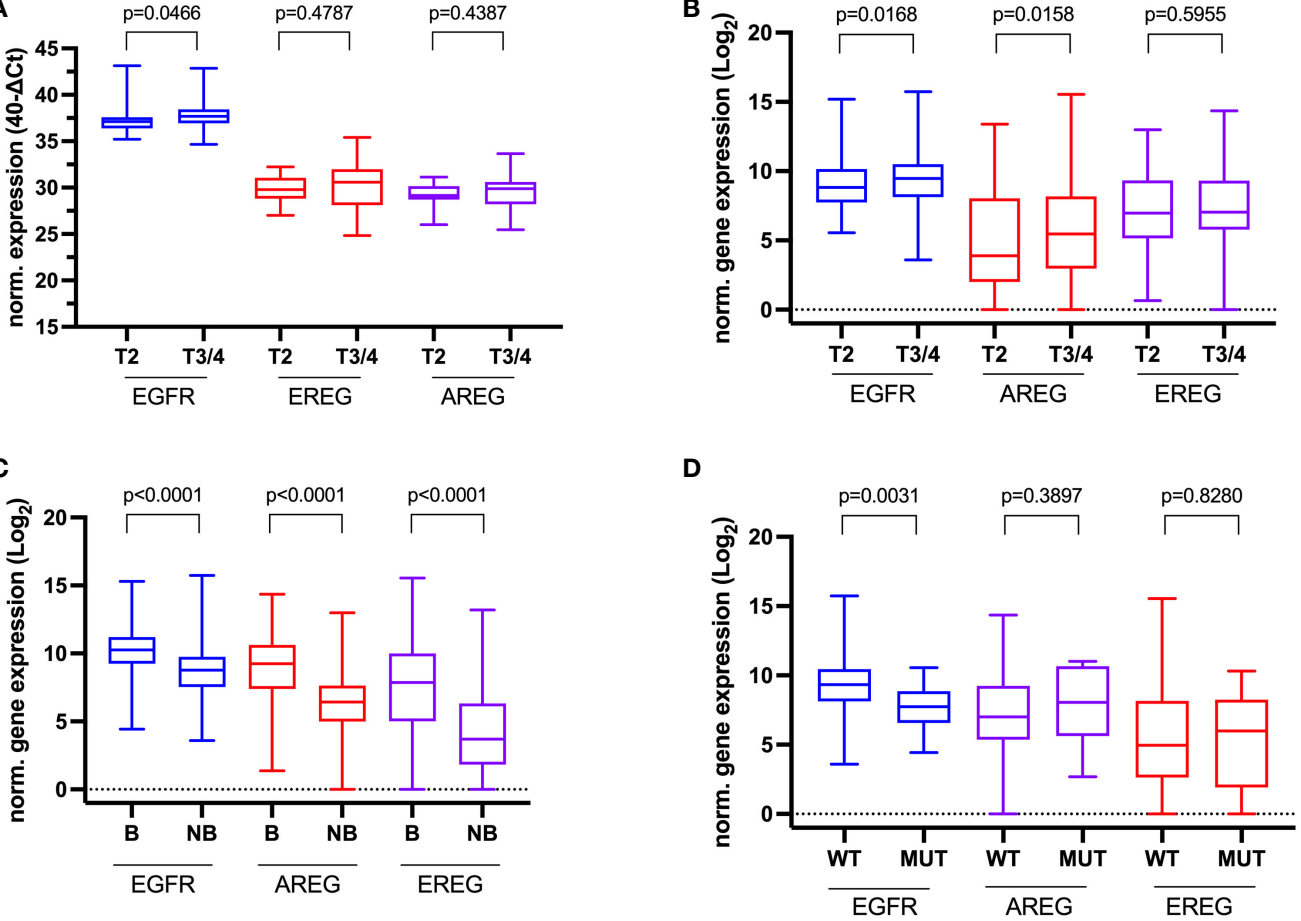
Association of clinicopathologic data with normalized gene expression. **(A)** Tumor stage in the Mannheim cohort, **(B)** Tumor stage in the TCGA cell 2017 MIBC cohort, **(C)** Molecular subtype (Basal **(B)** vs. Non-basal (NB) in the TCGA cell 2017 MIBC cohort, and **(D)** KRAS-Mutation status (wildtype (WT) vs. mutated (MUT) in the TCGA cell 2017 MIBC cohort.

In the TCGA cohort, T3/4 was correlated with a statistically significant increase in *EGFR* and *EREG* expression (*EGFR* p = 0.0176, *EREG* p = 0.0136, **Figure 4A**). However, this association could not be confirmed in the other three cohorts. Patients from the MA cohort with the presence of lymphovascular invasion (LVI) had a significantly lower *AREG* expression compared to patients without LVI (*AREG* 6.87 (range 5.37 – 8.69) vs. 7.78 (range 4.57 – 9.76); p = 0.0221, **Figure 4B**). Exploratory analysis of patients with a basal molecular subtype revealed a significantly higher gene expression for *EGFR* and both its ligands in patients with the basal molecular subtype (p < 0.0001 for each gene, **Figure 4C**). An exploratory analysis of the gene expression according to *KRAS* mutation status in the TCGA cohort showed a statistically significant higher expression of *EGFR* in patients with *KRAS* wildtype (vs. *KRAS* mutation; *EGFR* 9.35 (range 8.13 – 10.48) vs. 7.66 (range 6.59 – 8.53), p = 0.0002, **Figure 4D**). Further associations of the gene expressions with the investigated clinicopathologic characteristics are reported in **Supplementary Tables S4**, **S5**. In the Chungbuk cohort, N+ was associated with a significantly higher median *EGFR* expression (*EGFR* 8.37 (range 8.17 – 9.04) vs. 8.02 (range 7.24 – 10.32), p = 0.0485). In the MDACC cohort, female patients had a significantly higher *EGFR* expression (p = 0.0039) and patients with a basal molecular subtype had a significantly higher *EGFR* (p = 0.0053) and *EREG* expression (p = 0.0061).

### 3.4 Survival analyses

**Figure 5:**
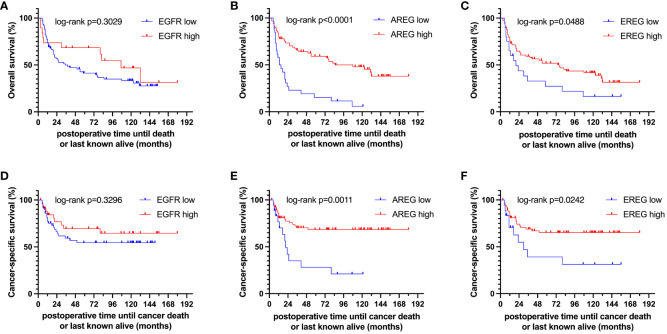
Kaplan-Meier curves of **(A–C)** overall survival (OS) and **(D–F)** cancer-specific survival (CSS) of *EGFR, AREG* and *EREG* in patients with MIBC from the Mannheim cohort.

**Figure 6:**
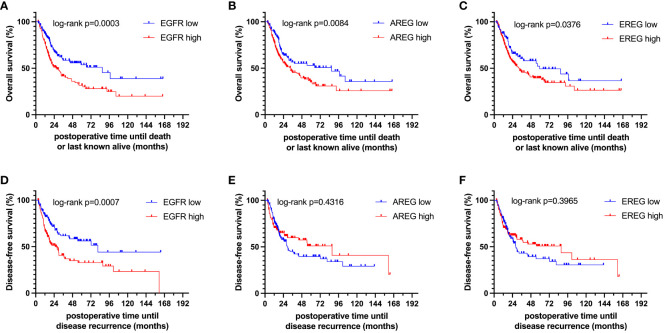
Kaplan-Meier curves of **(A–C)** overall survival (OS) and **(D–F)** disease-free survival (DFS) of *EGFR, AREG* and *EREG* in patients with MIBC from the TCGA cell 2017 MIBC cohort.

To evaluate the prognostic impact of *EGFR*, *AREG* and *EREG* expression on OS and CSS in the Chungbuk and MDACC, as well as OS, CSS and DFS in the MA cohort, and OS and DFS in the TCGA cohort, Kaplan-Meier analyses and cox proportional hazard ratios (HR) were used **Figures 5**, **6**. In the MA cohort, a high *AREG* expression was associated with a longer OS (n = 94, median survival (high vs. low) 85 vs. 14 months, p < 0.0001, **Figure 5B**) and CSS (n = 84, median survival (high vs low) undefined (since the probability of survival exceeds 50% at the most distant time point measured) vs. 21 months, p = 0.0011, **Figure 5E**). Like *AREG*, a high *EREG* expression was associated with improved OS (n = 94, median survival (high vs. low) 76 vs. 20 months, p = 0.0488, **Figure 5C**) and improved CSS (n = 84, median survival (high vs. low) undefined vs. 30 months, p = 0.0242, **Figure 5E**). No statistically significant data for DFS were observed in the MA cohort (data not shown). In the TCGA cohort, a high *EGFR*, *AREG* and *EREG* gene expression was associated with shorter OS for all three genes (n = 330, *EGFR*: median survival (high vs. low) 27 vs. 87 months, p = 0.0003; *AREG*: median survival (high vs. low) 33 vs. 87 months, p = 0.0084, *EREG*, median survival (high vs. low) 33 vs. 59 months, p = 0.0376, **Figures 6A–C**). The association of worse survival with a high gene expression was maintained on DFS for all three genes but only reached statistical significance for *EGFR* (*EGFR*: n = 258, median survival (high vs. low) 28 vs. 81 months, p = 0.0007, **Figures 6D, E**). In the Chungbuk cohort, a high *EGFR* expression was associated with worse OS (*EGFR*: n = 54, median survival (high vs. low) 10 vs. 66 months, p = 0.0008; **Supplementary Figure S4A**). Due to the small sample size in the MDACC MIBC cohort (n=38), the observed survival effects could not be adequately evaluated. Univariable analysis of the MA cohort revealed locally advanced T stage (T3/4) and N+ to be associated with shorter OS, while a high *AREG* expression (HR = 0.30, CI 0.18 – 0.52, p < 0.0001) was associated with longer OS (**Table 2**). A high *EREG* expression (HR = 0.57 CI 0.32 – 1.01, p=0.0538) was similarly associated with a trend for improved OS, but no statistical significance could be reached (**Table 2**). On multivariable analysis of OS in the MA cohort, advanced T stages and N+ were associated with a worse outcome, while *AREG* (HR = 0.35, CI 0.19 - 0.63, p = 0.0004) maintained its association with improved survival (**Table 2**). Regarding CSS in the MA cohort, univariable analysis showed T3/4 and N+ to be associated with shorter CSS while a high *AREG* (HR = 0.32, CI 0.15 – 0.66, p=0.0021) and *EREG* (HR = 0.43, CI 0.20 - 0.92, p=0.0296) expression correlated with improved CSS (**Table 3**). In the multivariable analysis, the *AREG* expression (HR = 0.42, CI 0.18 - 0.95, p = 0.0378) remained as the only significant predictor of better CSS (**Table 3**). In the univariable analysis of the TCGA cohort, age, T3/4, N+ and high *EGFR*, *AREG* and *EREG* gene expression (EGFR: HR = 1.81, CI 1.30 – 2.52, p = 0.0004; AREG: HR = 1.57, CI 1.12 – 2.20, p = 0.0090; EREG: HR = 1.48, CI 1.02 – 2.15, p = 0.0389) were significant prognostic factors for shorter OS (**Table 4**). Except for *EREG* this negative survival association was maintained for all of the variables included in the multivariable model (**Table 4**). Univariable analysis of DFS in the TCGA cohort showed worse DFS with T3/4, N+, *EGFR* amplification (HR = 2.49, CI 1.26 – 4.95, p = 0.0389) and a high *EGFR* expression (HR = 1.91, CI 1.31 – 2.80, p = 0.0008, **Table 5**). While the association of *EGFR* amplification with a shorter DFS could not withstand in the multivariable analysis, T3/4, N+ and a high *EGFR* gene expression (HR = 1.73, CI 1.14 – 2.62, p = 0.0094) remained significant independent factors for a worse DFS (**Table 5**). In the Chungbuk cohort, a higher *AREG* expression was associated with a trend towards better OS (HR = 0.45, CI 0.19 – 1.08, p = 0.0735) on univariable analysis and a significant predictor of better OS (HR = 0.31, CI 0.11 – 0.87, p = 0.0257) in the multivariable OS analysis (**Supplementary Table S6**).

**Table 2:** Uni- and multivariable cox regression analyses of gene expression and clinicopathological parameters regarding overall survival (OS) in patients with MIBC after radical cystectomy (RC) in the MA cohort.

Parameter	Univariable analysis		Multivariable analysis	
	P value	HR (95% CI)	P value	HR (95% CI)
Gender (female vs. male)	0.4606	1.24 (0.69 – 2.19)	–	–
Age (≥70 years vs. <70 years)	0.1489	1.48 (0.87 – 2.53)	0.0753	1.65 (0.95 – 2.88)
T stage (T3/4 vs. T2)	**0.0013**	2.93 (1.52 – 5.66)	**0.0065**	2.62 (1.31 – 5.25)
N stage (positive vs. negative)	**0.0051**	2.26 (1.28 – 3.99)	**0.0086**	2.27 (1.23 – 4.2)
*EGFR* copy number (AMP vs. NONAMP)	0.8689	0.94 (0.48 – 1.86)	–	–
*EGFR* expression (high vs. low)	0.3088	0.70 (0.36 – 1.39)		
*AREG* expression (high vs. low)	**<0.0001**	0.30 (0.18 – 0.52)	**0.0004**	0.35 (0.19 - 0.63)
*EREG* expression (high vs. low)	0.0538	0.57 (0.32 – 1.01)	0.7350	0.90 (0.48 – 1.67)

Significant p-values are highlighted in bold.

**Table 3:** Uni- and multivariable cox regression analyses of different parameters regarding cancer-specific survival (CSS) in patients with MIBC after RC in the MA cohort.

Parameter	Univariable analysis		Multivariable analysis	
	P value	HR (95% CI)	P value	HR (95% CI)
Gender (male vs. female)	0.4900	0.76 (0.35 – 1.65)		
Age (<70 years vs. ≥70 years)	0.9890	0.10 (0.49 – 2.03)		
T stage (T3/4 vs. T2)	**0.0195**	3.14 (1.20 – 8.21)	0.3258	1.34 (0.75 – 2.41)
N stage (positive vs. negative)	**0.0069**	2.85 (1.33 – 6.10)	0.2248	0.60 (0.26 – 1.37)
*EGFR* copy number (AMP vs. NONAMP)	0.2517	0.53 (0.18 – 1.57)		
*EGFR* expression (high vs. low)	0.33353	0.69 (0.33 - 1.47)		
*AREG* expression (high vs. low)	**0.0021**	0.32 (0.15 – 0.66)	**0.0378**	0.42 (0.18 - 0.95)
*EREG* expression (high vs. low)	**0.0296**	0.43 (0.20 - 0.92)	0.3993	1.41 (0.64 – 3.11)

Significant p-values are highlighted in bold.

**Table 4:** Uni- and multivariable cox regression analyses of different parameters regarding OS in patients of the TCGA cohort.

Parameter	Univariable analysis		Multivariable analysis	
	P value	HR (95% CI)	P value	HR (95% CI)
Gender (male vs. female)	0.3833	0.85 (0.60 – 1.22)		
Age (≥70 years vs. <70 years)	**0.0068**	1.57 (1.13 – 2.16)	**0.0052**	1.63 (1.16 – 2.29)
T stage (T3/4 vs. T2)	**0.0002**	2.12 (1.43 – 3.15)	**0.0168**	1.71 (1.10 – 2.65)
N stage (positive vs. negative)	**<0.0001**	2.25 (1.54 – 3.30)	**<0.0001**	2.04 (1.45 – 2.89)
*EGFR* copy number (AMP vs. NONAMP)	0.2378	1.50 (0.76 – 2.96)		
*EGFR* expression (high vs. low)	**0.0004**	1.81 (1.30 – 2.52)	**0.0150**	1.57 (1.09 – 2.26)
*AREG* expression (high vs. low)	**0.0090**	1.57 (1.12 – 2.20)	**0.0286**	1.60 (1.05 – 2.43)
*EREG* expression (high vs. low)	**0.0389**	1.48 (1.02 – 2.15)	0.8535	1.04 (0.66 – 1.66)

Significant p-values are highlighted in bold.

**Table 5:** Uni- and multivariable cox regression analyses of different parameters regarding DFS in patients with MIBC after RC in the TCGA cohort.

Parameter	Univariable analysis		Multivariable analysis	
	P value	HR (95% CI)	P value	HR (95% CI)
Gender (male vs. female)	0.5637	0.89 (0.60 – 1.33)		
Age (≥70 years vs. <70 years)	0.2828	1.22 (0.85 – 1.77)		
T stage (T3/4 vs. T2)	**<0.0001**	2.52 (1.61 – 3.92)	**0.0039**	2.08 (1.27 – 3.43)
N stage (positive vs. negative)	**<0.0001**	2.15 (1.54 – 3.02)	**0.0008**	1.95 (1.32 – 2.87)
*EGFR* copy number (AMP vs. NONAMP)	**0.0090**	2.49 (1.26 – 4.95)	0.1186	1.88 (0.85 – 4.14)
*EGFR* expression (high vs. low)	**0.0008**	1.91 (1.31 – 2.80)	**0.0094**	1.73 (1.14 – 2.62)
*AREG* expression (high vs. low)	0.4322	0.86 (0.58 – 1.26)		
*EREG* expression (high vs. low)	0.3972	0.85 (0.59 – 1.23)		

Significant p-values are highlighted in bold.

## 4 Discussion

In this study, the gene expression of *EGFR*, *AREG* and *EREG* in patients with MIBC, the association of gene expression with clinicopathologic variables of known prognostic impact in MIBC and the correlation of *EGFR*, *AREG* and *EREG* gene expression with survival and progression were investigated.

It was found that high *AREG* expression independently influenced the prediction of significantly longer OS and CSS in the MA (no significant survival associations for DFS) and longer OS in the Chungbuk cohort. In the TCGA cohort, a high *EGFR*, *AREG* and *EREG* expression were associated with worse OS and DFS. While this association was maintained for *EGFR* and *EREG* in the Chungbuk cohort, a high *EGFR* expression shifted from worse to better CSS prognosis in the Chungbuk cohort. The contradictory nature of these survival results in the three cohorts can partly be attributed to differences in analysis methods (qRT-PCR in the MA cohort, RNA-Seq in the TCGA cohort and microarray gene expression profiling in the Chungbuk cohort) and demographic baseline characteristics. We chose qRT-PCR in favor of IHC to analyze the gene expression, because the former method is free of inter-rater variability and proposed to be a more sensitive and unbiased method (23, 24). Additionally, patients’ treatments may further influence differences in gene expression profiles between individual cohorts. We compared the median gene expression of *EGFR*, *AREG* and *EREG* in patients with and without adjuvant chemotherapy in the MA cohort using the Mann-Whitney-Test, but could not find any statistically significant differences between both groups (data not shown).

Our observation that a high *AREG* expression was independently associated with better OS and CSS in the MA cohort is supported by data from Khambata-Ford et al., who found high expression levels of *AREG* and *EREG* on GeneChips and qRT-PCR to be associated with a significantly prolonged PFS under Cetuximab monotherapy (12). Correlation analysis further revealed *AREG* and *EREG* expression to be moderately (MA) and highly (TCGA) correlated with each other, which is in line with findings by Khambata-Ford et al. from mCRC and mostly attributable to colocalization of *AREG* and *EREG* on chromosome 4q13.3 (25). The observed weak correlation between *EGFR* and the two ligands could be explained by the ligand-receptor interaction, with EREG known to bind weaker to EGFR than other ligands but eliciting a stronger and prolonged EGFR activation (26, 27)

Biologically, elevated *AREG* and *EREG* expression have been postulated to stimulate an autocrine loop through EGFR leading Khambata-Ford et al. to hypothesize that these ligands are surrogate markers for an activated EGFR pathway, and potentially a positive feedback loop with EGFR (12). This hypothesis would be supported by our results of worse OS and DFS with a high expression of *AREG*, *EREG* and *EGFR* in the TCGA cohort.

As expected, *EGFR* gene expression was higher than that of its ligands in both cohorts and *EGFR* amplification events were associated with an increase in the expression of all three genes, while only *EGFR* was significantly differently expressed in the TCGA cohort. These findings correspond to the fact that, similar to CRC, activating mutations of *EGFR* in MIBC are rare, and *EGFR* expression is mainly regulated via *EGFR* amplification (12, 14).

Correlation with clinicopathologic data revealed advanced T stages T3/4 (TCGA: *EGFR* and *EREG*) and LVI (MA: *AREG*) to be the only variables associated with significant differences in gene expression. Further mechanistic studies are needed to ascertain if gene expression of *EGFR* and *EREG*/*AREG* truly drives local tumor growth, metastasis, and invasion or whether the observed differences are merely a bystander effect of the increased neoplastic microenvironment activity.

Interestingly, *EGFR*, although not statistically significant, was associated with better OS and CSS in the MA cohort, which corresponds to findings from mCRC showing no association of mRNA expression levels of *EGFR* and other *EGFR*-ligands (except for AREG and EREG) with disease control under Cetuximab (12).

Although a multitude of urinary and blood-based biomarkers exist to detect, monitor and control treatment response in NMIBC, survival prognosis for MIBC after RC is largely based on conventional imaging (CT and MRI scans). Since the gain of EGFR function is an established genomic event in the progression to MIBC, increased EGFR gene expression or amplification from FFPE tissue at the time of RC could be used as an additional marker for tumor aggressiveness besides histopathology and potentially serve as a biomarker (28). Smalley et al. used mass spectrometry to identify potential biomarkers on microparticles in the urine of patients with BC. They were able to show that 5 of the 8 detected proteins were associated with the EGFR pathway (29, 30). Other growth factors, such as the vascular endothelial growth factor A, which is part of the Oncuria® multiplex immunoassay (31), and the fibroblast growth factor 3 (FGFR3), which is part of the FDA-approved Uromonitor test® (32), are currently used in the context of urinary biomarker assays in clinical practice for NMIBC (33). While the above-mentioned markers were derived from urine, there is a clinical application for biomarkers evaluated by qRT-PCR of FFPE tissue samples, since FGFR3 mutations and FGFR2/3 gene fusions, which are currently used to guide the use of the pan-FGFR-inhibitor Erdafitinib for metastatic BC, are assessed using qRT-PCR of FFPE tissue samples (34).

Limitations of this study include its retrospective nature, varying gene expression detection and normalization methods between the MA and the TCGA cohort. Comparisons to other trials are limited through our endpoint selection of OS, CSS and DFS contrary to PFS and treatment response to anti-EGFR therapy. However, to our knowledge this is the first trial to date to assess clinicopathologic and survival associations of *AREG* and *EREG* in patients with MIBC.

Ultimately, the different findings between the MA and TCGA cohorts will need to be further investigated, as differences in used methodology, the existence of splice variants and differences in cohorts are potential biases to a validation. Further *in vitro* studies are needed to examine the nature of the EGFR-AREG/EREG relationship at a molecular level in MIBC.

AREG and EREG are promising prognostic markers in MIBC. Validation in the TCGA and Chungbuk cohort shows diverging survival results. Further *in vitro* studies at the molecular level are needed to explore the nature of the EGFR-AREG/EREG interaction and its potential impact on BC cancer biology and survival.

## Data availability statement

The datasets presented in this study can be found in online repositories. The names of the repository/repositories and accession number(s) can be found in the article/**Supplementary Material**.

## Ethics statement

The studies involving humans were approved by review board 2 of the University of Heidelberg under the number 2015- 549N-MA. The studies were conducted in accordance with the local legislation and institutional requirements. The participants provided their written informed consent to participate in this study.

## Author contributions

DU: Conceptualization, Data curation, Formal analysis, Investigation, Methodology, Visualization, Writing – original draft, Writing – review & editing. BT: Data curation, Writing – review & editing. AF: Data curation, Writing – review & editing. DJ: Data curation, Writing – review & editing. ZP: Data curation, Writing – review & editing. FS: Formal analysis, Writing – review & editing. MM: Writing – review & editing. PN: Writing – review & editing. TW: Writing – review & editing. PE: Conceptualization, Supervision, Writing – review & editing. KN: Conceptualization, Data curation, Formal analysis, Investigation, Writing – original draft, Writing – review & editing.
