# Supplementary material for: Prognostic significance of EGFR, AREG and EREG amplification and gene expression in muscle invasive bladder cancer
Source: Front Oncol. 2024 May 28;14:1370303. doi: 10.3389/fonc.2024.1370303 (PMC11168109; doi:10.3389/fonc.2024.1370303)
Supplement: Supplementary file 5 [file DataSheet_1.docx]

Supplementary Material

**Prognostic Significance of EGFR. AREG and EREG Amplification and Gene Expression in Muscle Invasive Bladder Cancer**

**Daniel Uysal^1^. Blerta Thaqi^1^. Alexander Fierek^1^. David Jurgowski^1^. Zoran V Popovic^2^, Fabian Siegel^3^, Maurice Stephan Michel^1^, Philipp Nuhn^4^, Thomas Stefan Worst^1^, Philipp Erben^1^, Katja Nitschke^1*^**

^1^Urologic Research Center, Department of Urology and Urosurgery, Medical Faculty Mannheim, University of Heidelberg, Mannheim, Germany

^2^ Institute of Pathology, Medical Faculty Mannheim, University of Heidelberg, Mannheim, Germany

^3^ Department of Biomedical Informatics at the Center for Preventive Medicine and Digital Health, Medical Faculty of Mannheim, University of Heidelberg, Mannheim, Germany

^4^ Department of Urology, Universitätsklinikum Schleswig-Holstein (UKSH), Campus Kiel, Kiel, Germany

*** Correspondence:**Katja Nitschke PhD

Department of Urology and Urosurgery,

University Medical Centre Mannheim (UMM), Medical Faculty Mannheim of the University of Heidelberg

Theodor-Kutzer-Ufer 1-3, 68167 Mannheim, Germany
Katja.Nitschke@medma.uni-heidelberg.de

# Supplementary Data

Supplementary raw data Mannheim

Supplementary raw data TCGA

Supplementary raw data Chungbuk

Supplementary raw data MDACC cohort

# Supplementary Figures and Tables

## Supplementary Figures


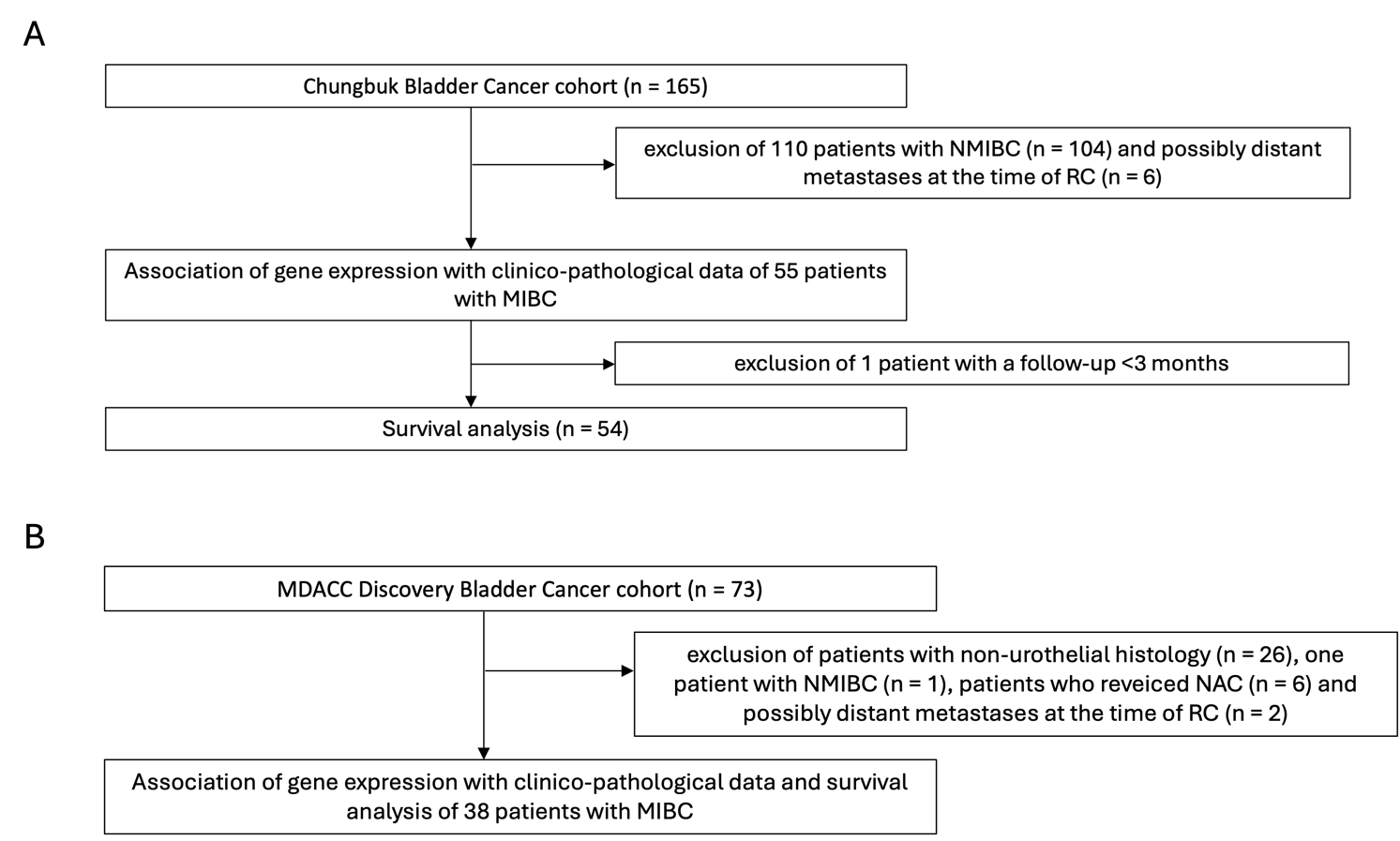


**Supplementary Figure S1.** REMARK flow diagram of the exclusion criteria of the third and forth study cohorts based on the Chungbuk cohort and the MDACC cohort. NMIBC: Non–muscle Invasive Bladder Cancer; RC: Radical Cystectomy; NAC: neoadjuvant chemotherapy.

**Supplementary Figure S2.** Gene expression of *AREG*, *EREG* and *EGFR* according to amplification status of EGFR (AMP (amplification) vs. NO (no amplification)). A. Data from the TCGA cohort. B. Data from the MA cohort.

**Supplementary Figure S3**. Spearman coefficient correlation analysis of *AREG*, *EREG* and *EGFR.* A – C: TCGA cohort. A. Correlation of *AREG* and *EREG, B.* Correlation of *AREG* and *EGFR*, C. Correlation of *EREG* and EGFR; D – F. MA cohort. D. Correlation of *AREG* and *EREG,* E*.* Correlation of *AREG* and *EGFR*, F. Correlation of *EREG* and EGFR.

**
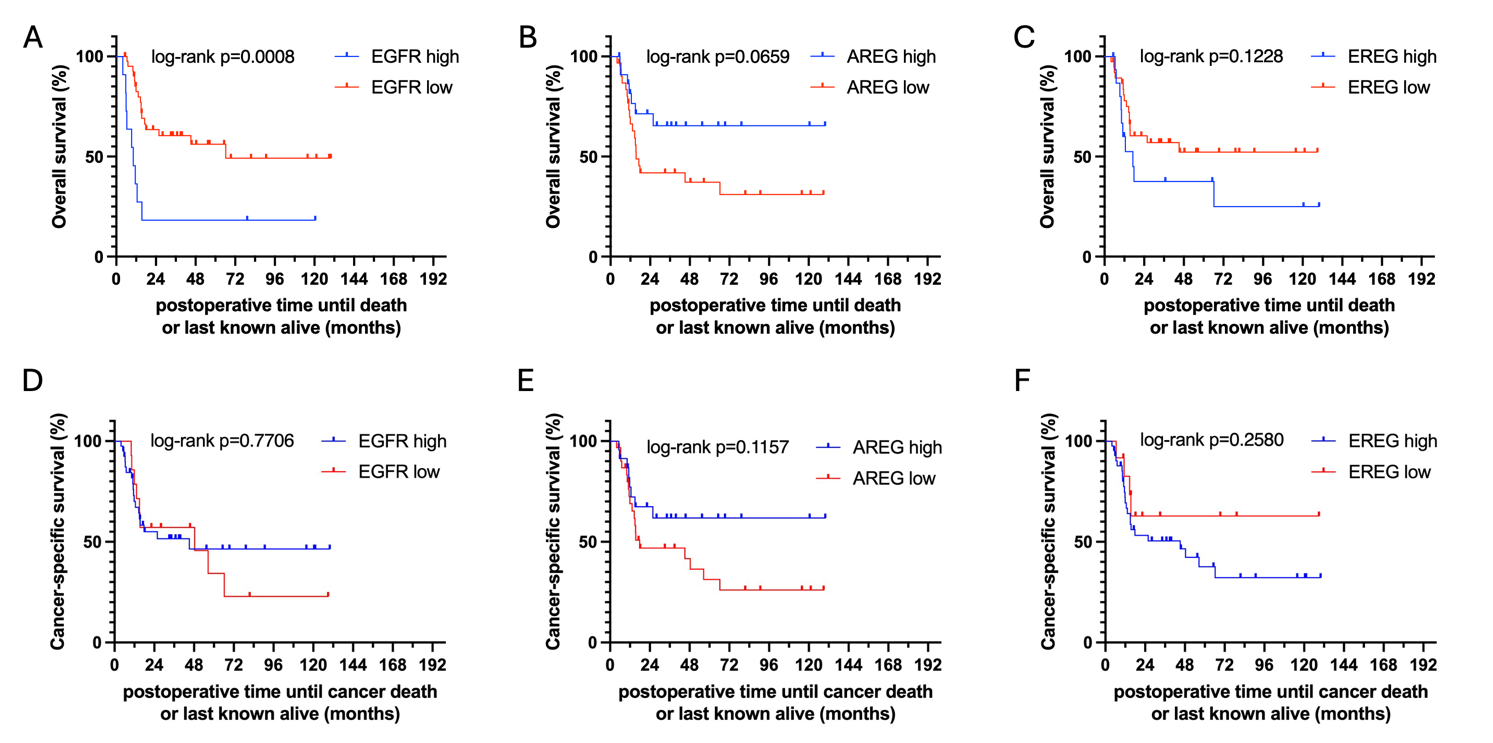
**

**Supplementary Figure S4**. Kaplan-Meier curves of (A – C) overall survival (OS) and (D – F) cancer-specific survival (CSS) of *EGFR, AREG* and *EREG* in patients with MIBC from the Chungbuk cohort.

## Supplementary Tables

Supplementary Table S1. Primers and probes used in the study.

| **Primer/Probe** | **Nucleotide sequence** | **Melt temperature Tm (°C)** |
| --- | --- | --- |
| AREG Forward | CAGTAACATGCAAATGTCAGCAAG | 59.3 |
| AREG Reverse | GCAGACATAAAGGCAGCTATGG | 60.3 |
| AREG Probe | [FAM]-GATAAACTACTGTCAATCA TGCTGTGAGTTTTCATGG-[BHQ1] | 68.2 |
| EREG Forward | GCACAGCTTTAGTTCAGACAGAAG | 61 |
| EREG Reverse | GTATAACCCACTTCACACCTGC | 60.3 |
| EREG Probe | [FAM]-TCATGTCCACCAGATAGATGCA  CTGTCCATG-[BHQ2] | 68.2 |
| EGFR Forward | CGCAAGTGTAAGAAGTGCGAA | 56.8 |
| EGFR Reverse | CGTAGCATTTATGGAGAGTGAGTCT | 56.1 |
| EGFR Probe | [FAM]-CCTTGCCGCAAAGTGTGTAACG  GAAT-[BHQ2] | 62.7 |
| CALM2 Reverse | GAGCGAGCTGAGTGGTTGTG | 61.4 |
| CALM2 Reverse | AGTCAGTTGGTCAGCCATGCT | 59.8 |
| CALM2 Probe | [FAM]-TCGCGTCTCGGAAACCGGTAGC-[BHQ1] | 65.8 |

Supplementary Table S2. Taqman Copy Number Assay used in the study.

| **Gen** | **Taqman Copy Number Assay Name** | **Location** | **Cytoband** | **Amplicon Length** |
| --- | --- | --- | --- | --- |
| EGFR | Hs00711969_cn | Chr.7:55198875 on GRCh38 | 7p11.2 | 83 |
| RNAse P (Reference Assay) |  | Chr.14:20343370 | 14q11.2 | 87 |

Supplementary Table S3. Spearman coefficient correlation analysis of *AREG*, *EREG* and *EGFR* in the Chungbuk and MDACC cohorts. Significant values are shown in bold.

|  | Variable | Variable | **Spearman ρ** | **Prob>\|ρ\|** |
| --- | --- | --- | --- | --- |
| Chungbuk | AREG | EGFR | 0.0410 | 0.7664 |
|  | EREG | EGFR | 0.1309 | 0.3408 |
|  | EREG | AREG | 0.0091 | 0.9472 |
| MDACC | AREG | EGFR | -0.0141 | 0.9330 |
|  | EREG | EGFR | 0.3457 | **0.0335** |
|  | EREG | AREG | 0.0594 | 0.7231 |

Supplementary Table S4. Clinicopathologic associations of the gene expression of *EGFR, AREG* and *EREG* in the TCGA cohort. For each variable median gene expression, range (10^th^ percentile – 90^th^ percentile) and the p value from the Mann-Whitney-U test are provided. Significant values are shown in bold.

| Variable | ***EGFR*** | | ***AREG*** | | ***EREG*** | |
| --- | --- | --- | --- | --- | --- | --- |
|  | Gene expression | P value | Gene expression | P value | Gene expression | P value |
| **LVI** |  |  |  |  |  |  |
| No | 9.1230 (7.037 – 11.196) | 0.7177 | 7.7810 (4.571 – 11.492) | 0.0221 | 4.9450 (1.1762 – 10.69) | 0.9639 |
| Yes | 9.4125 (6.4757 – 11.696) |  | 6.8715 (4.0476 – 10.601) |  | 4.8975 (1.7828 – 9.9643) |  |
| **N stage** |  |  |  |  |  |  |
| N0 | 9.1155 (6.6812 – 11.37) | 0.0527 | 7.1955 (3.954 – 10.915) | 0.6297 | 4.8635 (1.2466 – 10.378) | 0.6117 |
| N+ | 9.6290 (7.1043 – 11.75) |  | 6.9140 (3.6414 – 11.039) |  | 5.1620 (1.7326 – 10.214) |  |
| **T stage** |  |  |  |  |  |  |
| T2 | 8.8420 (6.5126 – 11.216) | **0.0175** | 6.9270 (2.992 - 9.146) | 0.3111 | 4.089 (0.88438 - 10.472) | **0.0136** |
| T3/4 | 9.4825 (6.9355 - 11.75) |  | 7.1025 (3.96 - 10.885) |  | 5.4695 (1.7175 - 10.09) |  |
| **Gender** |  |  |  |  |  |  |
| Female | 9.3970 (6.9194 - 11.694) | 0.6247 | 7.3070 (3.8194 - 10.662) | 0.8218 | 5.3850 (1.5126 - 10.372) | 0.3121 |
| Male | 9.1925 (6.7215 - 11.59) |  | 6.9805 (3.595 - 10.91) |  | 4.7980 (1.3 - 10.385) |  |
| **Age** |  |  |  |  |  |  |
| <70 | 9.1720 (6.8896 - 11.56) | 0.9216 | 7.0010 (3.8194 - 10.614) | 0.4792 | 4.4750 (1.3306 - 10.072) | 0.1649 |
| ≥70 | 9.3540 (6.7215 - 11.735) |  | 7.1755 (3.655 - 11.125) |  | 5.1625 (1.4915 - 10.525) |  |

Supplementary Table S5. Clinicopathologic associations of the gene expression of *EGFR. AREG* and *EREG* in the Mannheim cohort. For each variable median gene expression, range (10^th^ percentile – 90^th^ percentile) and the p value from the Mann-Whitney-U test are provided.

| Variable | *EGFR* |  | *AREG* |  | *EREG* |  |
| --- | --- | --- | --- | --- | --- | --- |
|  | Gene expression (CT value) | P value | Gene expression (CT value) | P value | Gene expression (CT value) | P value |
| **LVI** |  |  |  |  |  |  |
| No | 37.46 (36.29 - 39.25) | 0.4506 | 29.68 (26.91 - 30.98) | 0.9307 | 29.79 (26.87 - 32.3) | 0.7295 |
| Yes | 37.63 - 39.69) |  | 29.34 (27.03 - 30.98) |  | 29.82 (27.17 - 32.96) |  |
| **N stage** |  |  |  |  |  |  |
| N0 | 37.54 (36.32 - 39.35) | 0.8927 | 29.62 (27.05 - 31.04) | 0.2000 | 29.94 (27.25 - 32.99) | 0.2101 |
| N+ | 37.60 (36.64 - 39.78) |  | 28.76 (26.68 - 30.88) |  | 29.67 (24.65 - 32.59) |  |
| **T stage** |  |  |  |  |  |  |
| T2 | 37.34 (36.22 - 38.70) | 0.1101 | 29.17 (26.36 - 31.03) | 0.8773 | 29.80 (28.18 - 32.24) | 0.3588 |
| T3/4 | 37.61 (36.57 - 39.55) |  | 29.51 (27.05 – 30.97) |  | 29.78 (26.87 – 33.09) |  |
| **Gender** |  |  |  |  |  |  |
| Female | 37.45 (36.79 - 38.76) | 0.2569 | 29.79 (27.08 - 30.72) | 0.2651 | 31.05 (26.98 - 33.47) | 0.1282 |
| Male | 37.54 (36.27 - 39.19) |  | 29.19 (26.95 - 30.82) |  | 29.70 (26.94 - 32.24) |  |
| **Age** |  |  |  |  |  |  |
| <70 | 37.42 (36.24 - 39.67) | 0.3165 | 29.75 (26.89 - 30.84) | 0.7759 | 30.42 (26.67 - 32.24) | 0.7422 |
| ≥70 | 37.62 (36.37 - 39.40) |  | 29.29 (27.04 – 31.11) |  | 29.67 (26.99 - 33.16) |  |

Supplementary Table S6. Uni- and multivariable cox regression analyses of gene expression and clinicopathological parameters regarding overall survival (OS) and cancer-specific survival (CSS) in patients with MIBC in the Chungbuk cohort. Significant values are shown in bold.

|  | **Overall survival** | | | | **Cancer-specific survival** | | | |
| --- | --- | --- | --- | --- | --- | --- | --- | --- |
| **Parameter** | Univariable analysis | | Multivariable analysis | | Univariable analysis | | Multivariable analysis | |
|  | P value | HR (95% CI) | P value | HR (95% CI) | P value | HR (95% CI) | P value | HR (95% CI) |
| Gender  (male vs. female) | 0.0518 | 2.29 (0.99 – 5.30) | 0.4746 | 1.38 (0.57 – 3.30) | 0.0247 | 2.51 (1.12 – 5.61) | 0.1513 | 1.84 (0.80 – 4.24) |
| Age  (≥70 years vs. <70) | **0.0001** | 5.17 (2.21 – 12.05) | **0.0001** | 6.71 (2.56 – 17.59) | **<0.0001** | 6.15 (2.63 – 14.40) | **0.0001** | 5.66 (2.33 – 13-76) |
| T stage  (T2 vs. T3/4) | **0.0431** | 2.28 (1.03 – 5.08) | 0.3929 | 1.51 (0.59 – 3.88) | **0.0084** | 2.97 (1.32 – 6.69) | 0.1091 | 2.13 (0.85 – 5.35) |
| N stage  (N0 vs. N+) | 0.1321 | 2.16 (0.79 – 5.86) | 0.6688 | 1.30 (0.39 – 4.37) | **0.0055** | 3.62 (1.46 – 8.97) | 0.0533 | 2.55 (0.99 – 6.60) |
| *EGFR*  (high vs. low) | **0.0017** | 3.69 (1.63 – 8.37) | **0.0259** | 3.34 (1.16 – 9.64) | 0.7710 | 0.89 (0.40 - 1.98) |  |  |
| *AREG*  (high vs. low) | 0.0735 | 0.45 (0.19 – 1.08) | **0.0257** | 0.31 (0.11 - 0.87) | 0.1226 | 0.52 (0.23 – 1.19) | 0.1431 | 0.51 (0.21 – 1.25) |
| *EREG*  (high vs. low) | 0.1291 | 1.85 (0.84 – 4.08) | 0.0518 | 2.68 (0.99 – 7.24) | 0.2658 | 1.83 (0.63 – 5.29) |  |  |
